# Molecular docking *versus* co-folding for protein–peptide pose prediction: effects of protein flexibility and training set overlap

**DOI:** 10.1039/d6dd00242k

**Published:** 2026-09-11

**Authors:** Mark Fonteyne, Nada Badr, Peter J. K. Kuppen, Alexander L. Vahrmeijer, Gerard J. P. van Westen, Willem Jespers

**Affiliations:** a Department of Surgery, Leiden University Medical Center 2333 ZA Leiden The Netherlands m.fonteyne@lumc.nl n.badr@lumc.nl p.j.k.kuppen@lumc.nl a.l.vahrmeijer@lumc.nl; b Medicinal Chemistry, Leiden Academic Centre of Drug Research (LACDR), Leiden University 2333 CC Leiden The Netherlands gerard@lacdr.leidenuniv.nl; c Department of Pharmacy, University of Groningen 9713 AV Groningen The Netherlands w.jespers@rug.nl

## Abstract

Synthetic peptides are increasingly important as therapeutic and diagnostic agents due to their high specificity, ease in synthesis, and suitability for targeting protein interfaces. However, accurate prediction of peptide binding poses remains a major challenge, particularly for conformationally flexible protein targets and *de novo* designed peptides. Deep learning-based co-folding methods, including AlphaFold-Multimer (AFM), AlphaFold3 (AF3), and Boltz-2, offer an alternative to conventional peptide docking by simultaneously predicting protein–peptide complex structures without requiring predefined binding conformations. In this study, we systematically benchmarked these three co-folding methods against two peptide-optimized molecular docking methods, AutoDock-CrankPep (ADCP) and HADDOCK, using the PepPro dataset. This established dataset contains conformationally flexible protein–peptide complexes that are represented in the training sets of all co-folding methods. To assess the generalization performance of the co-folding methods, an independent test set was constructed classified by its level of redundancy with the training sets. The benchmark results showed that co-folding methods outperformed docking approaches in pose prediction accuracy and ranking performance when related complexes were represented in the training set. In the absence of such representation, however, HADDOCK outperformed all evaluated co-folding methods. Boltz-2 achieved the highest accuracy on the PepPro dataset but showed reduced generalization on non-redundant complexes, indicating strong dependence on training-set coverage. In contrast, AFM and AF3 demonstrated more robust generalization and maintained consistent performance on independent protein–peptide complexes, highlighting improved suitability for *de novo* peptide binder design. Furthermore, extensive analysis of scoring metrics revealed that interface predicted template modelling (ipTM) provides strong early enrichment performance, while peptide-specific predicted local distance difference test (pLDDT) correlates closely with structural accuracy, suggesting complementary value for candidate prioritization. These findings demonstrate that robust peptide-binder prediction requires careful consideration of training-set redundancy, target conformational flexibility, and complementary confidence metrics when selecting computational methods for *de novo* candidate prioritization.

## Abstract 2

Synthetic peptides are increasingly important as therapeutic and diagnostic agents due to their high specificity, ease in synthesis, and suitability for targeting protein interfaces.

## Graphical abstract

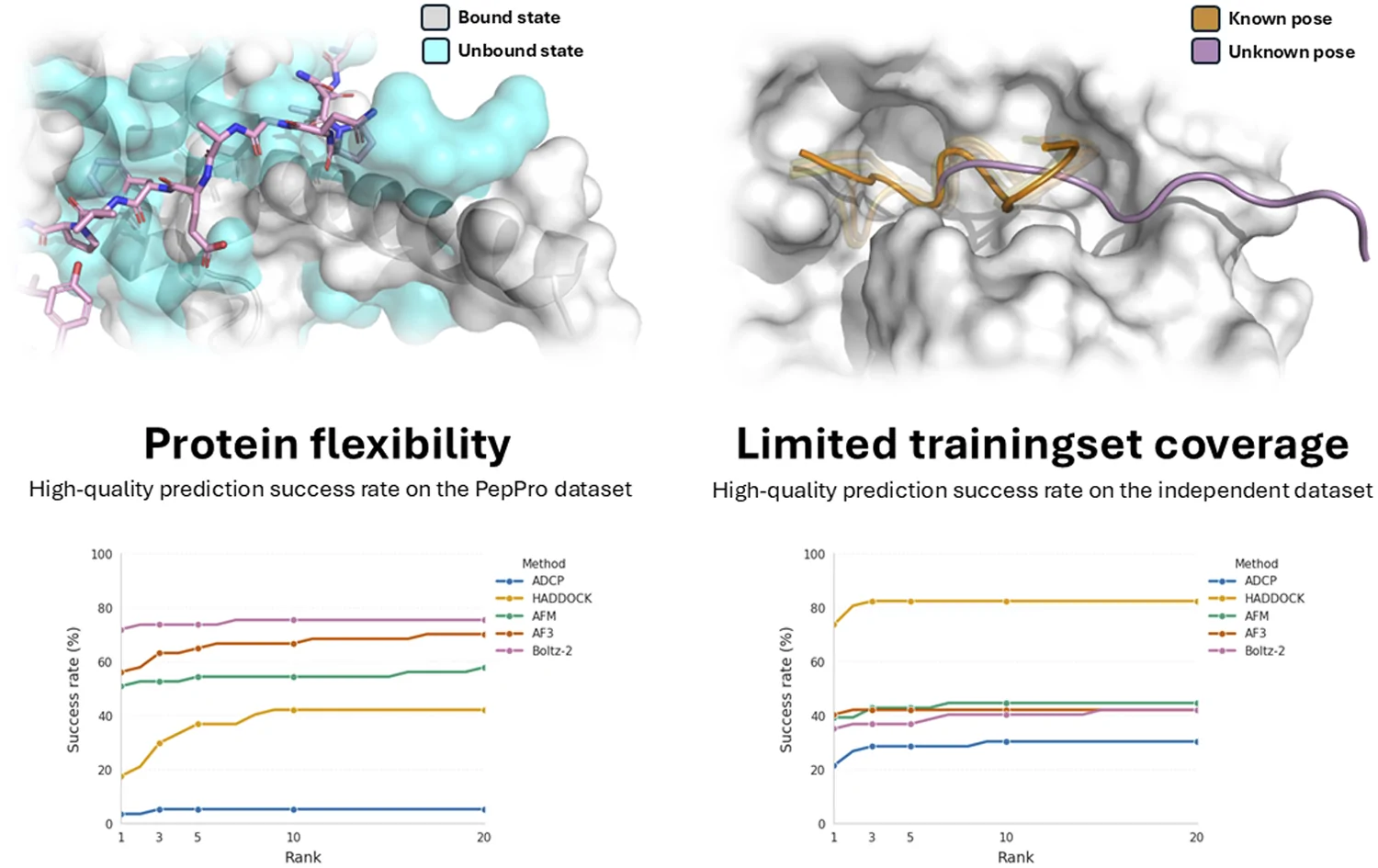

## Introduction

Recently, an increasing number of peptides have emerged as promising therapeutics owing to their target specificity, strong binding affinity, and cost-effective production.^1–3^ Therapeutic peptides are usually classically applied to act as hormones, growth factors, neurotransmitters, ion channel ligands, with one of the most well-known examples being the glucagon-like peptides 1 (GLP-1's) antidiabetics like semaglutide and liraglutide.^4^

Beyond therapeutic applications, peptides are increasingly explored in molecular imaging due to their high affinity and specificity for disease-associated biomarkers, including overexpressed cell-surface proteins. When conjugated to imaging moieties such as near-infrared fluorophores or radiolabels, peptide-based probes enable sensitive and selective visualization of target tissues, supporting applications such as fluorescence-guided surgery (FGS) and positron emission tomography (PET).^5^ Although antibodies currently dominate clinical imaging approaches, peptides offer important advantages, including smaller size (0.5–5 kDa), less complicated production, and rapid tissue penetration, while maintaining high target specificity.^1,2,6^ For example, cyclic RGD peptides selectively bind integrin subtypes such as αvβ3 and αvβ6, enabling targeted imaging of tumour tissues with elevated integrin expression while remaining compatible with standard GMP production.^7^

Despite the growing success of peptide therapeutics and diagnostics, the identification and optimization of high-affinity peptide binders remain a major challenge. Traditionally, the discovery of such peptides relied on labour-intensive and costly screening techniques, such as phage display, as conventional methods were unable to efficiently explore the vast peptide sequence space.^8^

To improve efficiency and reduce costs, computational approaches such as physics-based docking were developed to predict peptide binding through several interaction potential scoring functions before experimental validation.^9,10^ These methods provide a computationally accessible alternative, but they typically require either large, extensively curated experimental datasets or databanks to identify potential peptide hits or clearly defined peptide templates to reliably guide novel designs.^11,12^ Furthermore, they often struggled to accurately model the highly dynamic and flexible nature of protein–peptide interactions.^13^ In particular, most rely on static protein structures and may perform poorly when targets exhibit significant conformational heterogeneity.

To address these limitations, deep learning-based co-folding approaches have recently emerged that do not require predefined protein conformations.^14–16^ Rather than relying on physics-based scoring functions, these methods predict the structural folding of the protein and the peptide within a complex and evaluate model confidence based on internal consistency metrics. This makes them particularly powerful when conformational variability or limited structural data hinder traditional docking approaches. However, co-folding methods strongly depend on learned representations derived from their training data and multiple sequence alignment (MSA) information, which may introduce biases and limit generalization to out-of-distribution systems.^17^ Consequently, they can exhibit structural hallucinations, inaccurate side-chain geometries, and challenges in discriminating near-native from non-native poses.

AlphaFold-Multimer (AFM) was among the first approaches capable of folding a fully flexible peptide into a fully flexible protein and is still widely used in *de novo* peptide design pipelines.^18^ More recently, AlphaFold3 (AF3) and Boltz-2 have further advanced co-folding capabilities into the area of small molecules. AF3 transitioned from the invariant point attention (IPA)-based architecture of its predecessor to a conditional diffusion-based model operating at the all-atom level.^19^ This architectural shift improved performance and enables confidence prediction at atomic resolution. Boltz-2 builds upon AF3's architecture but introduces a physics-inspired steering mechanism called “Boltz-steering” during inference to reduce structural hallucinations and correct non-physical features such as steric clashes, distorted bond geometries, and non-planar aromatic systems.^20^ More importantly, Boltz-2 extends the capabilities of AF3 by providing a dedicated affinity prediction module. However, its validation primarily on small-molecule ligands and its atom-count restriction limit its applicability to larger peptides. These newer methods increasingly address small-molecule and atomic-level geometry challenges, expanding their applicability in structure prediction.

For small molecules, the effects of apo-to-holo receptor conformational changes on pose-prediction performance have been investigated in recent studies, although direct comparisons between molecular docking and co-folding methods on matched experimental apo–holo datasets remain limited.^21,22^ Similarly, although peptide co-folding methods have been compared with conventional peptide-docking approaches, their relative performance has not yet been systematically assessed using an apo–holo benchmark stratified according to the extent of experimentally observed receptor conformational change. Recent studies have attempted to characterize conformational variation using molecular dynamics simulations.^23,24^ However, these simulations are not equivalent to conformational changes derived from matched experimental apo and holo structures. To our knowledge, no study has directly benchmarked peptide-docking and co-folding methods using a dataset stratified by experimentally determined apo-to-holo receptor conformational changes.

To address this gap, this study evaluates two molecular docking methods, AutoDock-CrankPep (ADCP) and HADDOCK, together with the three co-folding methods AFM, AF3, and Boltz-2, for their peptide pose prediction performance on conformationally flexible protein–peptide complexes. ADCP and HADDOCK are two state-of-the-art docking platforms that incorporate peptide flexibility and experimental restraints to model peptide binding. ADCP leverages adaptive conformational sampling using “CRANKITE”, guided by a probabilistic scoring function similar to that of AutoDock Vina, to efficiently explore diverse peptide binding poses.^25^ HADDOCK applies an information-driven protocol integrating experimental or predicted restraints and includes limited protein flexibility through short molecular dynamics simulations.^26,27^ While both methods are well-suited for protein–peptide docking approaches as suggested by Weng *et al.*, they remain dependent on accurate static protein structures and may struggle with highly flexible or conformationally heterogeneous systems.^28^

The five methods were evaluated on the PepPro dataset, a non-redundant protein–peptide complex structural dataset comprising peptides ranging from 5 to 30 residues.^29^ The PepPro database contains protein–peptide complex structures with corresponding unbound protein structures, thereby providing matched experimentally determined holo and apo receptor states and enabling quantification of conformational changes upon peptide binding. This dataset therefore allowed us to evaluate the sensitivity of the methods to experimentally observed receptor conformational changes.

However, the PepPro dataset was constructed in 2019, and its structures fall within the training-set cut-off dates of the evaluated co-folding models. The three co-folding models may therefore have been trained on these specific structures, potentially inflating performance estimates and obscuring their true generalization capacity. To complement the conformational-change analysis and systematically assess potential training-data leakage, we constructed a novel, temporally separated external validation dataset containing protein–peptide complexes released after the respective model training cut-off dates. A recent study evaluating the generalization of protein-small-molecule co-folding methods showed that commonly used sequence-similarity thresholds, such as 40% sequence identity, were insufficient to reliably assess model generalization.^30^ Therefore, in addition to applying peptide-sequence similarity thresholds, our independent dataset was constructed using a purely structure-based comparison of the complex components, independent of sequence identity. This structural non-redundancy criterion enabled us to assess whether model performance reflected generalization or was influenced by three-dimensional structural similarity to related training examples. The validation set was further divided according to different levels of peptide-sequence and complex-structure redundancy, allowing model performance to be evaluated as similarity to potentially related training examples was progressively reduced. Together, these complementary evaluations assess both the effect of receptor conformational changes and the potential influence of training-data leakage. This distinction is particularly relevant for *de novo* peptide design, for which reliable predictive performance on previously unseen peptides and protein–peptide complexes is critical.

**Fig. 1:**
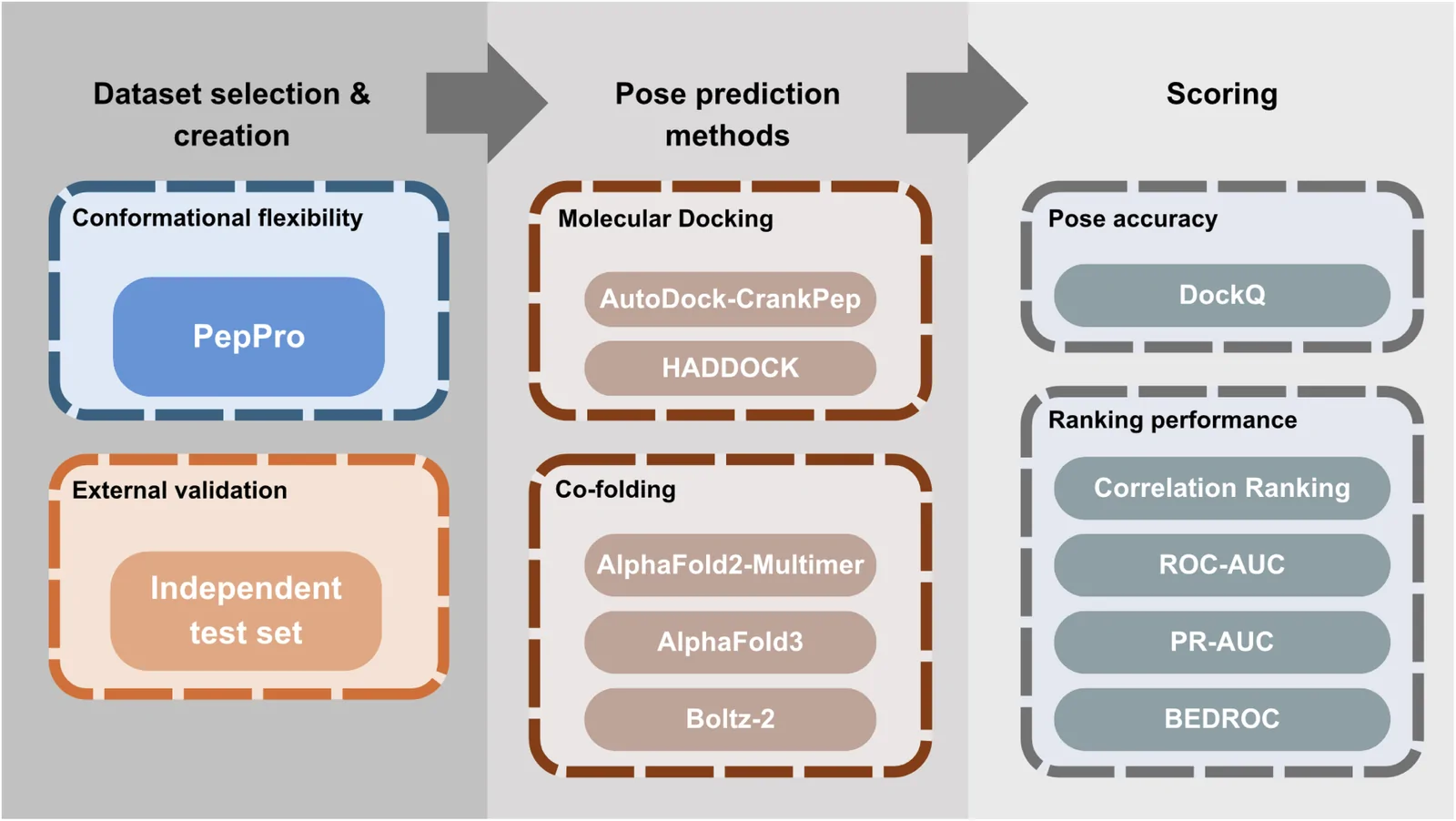
Benchmarking workflow of molecular docking and co-folding methods on the conformationally flexible PepPro dataset and a curated independent test set. Two peptide docking methods, AutoDock-CrankPep and HADDOCK, as well as three co-folding approaches, AlphaFold-Multimer, AlphaFold3, and Boltz-2, were employed to evaluate their pose prediction performance. The PepPro dataset, comprising 57 conformationally flexible protein–peptide complexes, was used to evaluate pose prediction performance on complexes with conformationally distinct apo and holo states. An independent test set, comprising 57 external validation protein–peptide complexes, was further employed to evaluate both the pose prediction accuracy and generalisation capability of the methods. Performance was quantified using five metrics: pose accuracy, correlation-based ranking, receiver operating characteristic area under the curve (ROC-AUC), precision-recall area under the curve (PR-AUC), and Boltzmann-enhanced discrimination of receiver operating characteristic (BEDROC).

Beyond evaluating structural pose accuracy, we analysed ranking performance and early enrichment behaviour to determine their suitability for realistic peptide design workflows, where only a limited number of candidates can be experimentally tested. Furthermore, we investigated the predictive value of internal metrics, including docking scores, interface predicted template modelling (ipTM) and peptide-specific predicted local distance difference test (pLDDT), to assess their ability to discriminate near-native from non-native poses and to prioritize high-quality predictions. Method robustness was investigated in the presence and absence of training-set overlap to assess generalization capacity. The overview of this study is depicted in Fig. 1.

## Materials & methods

### Conformational-dependency dataset

The PepPro dataset was used for benchmarking the performance of the docking methods on highly conformational complexes.^29^ The dataset was obtained from the Zou Laboratory website at the University of Missouri, and contains in total 89 non-redundant experimentally determined protein–peptide complex structures. The dataset was accessed on 14 February 2024. From the PepPro dataset, 57 protein–peptide complex structures were selected due to corresponding apo-state (unbound) protein structures availability. The holo-state proteins were used to assign the binding motifs for the physics-based docking methods, based on the peptide bound within. The protein conformational changes upon peptide binding were reported with an interface root mean square deviation based on backbone atoms (I-RMSD_b). Xu *et al.*^29^ defined the conformational changes as “close to bound structures” with an I-RMSD_b value smaller than 1.0 Å. Significant conformational changes were classified as 1.0 Å ≤ I-RMSD_b < 2.0 Å, and dramatic conformational changes were classified as I-RMSD_b ≥ 2.0 Å.

### Independent dataset

To assess the performance of the co-folding methods on an external validation dataset, a novel dataset was constructed that did not contain any protein–peptide complexes present in the internal training sets of the three co-folding methods. The RCSB Protein Data Bank (RCSB PDB) was used to extract experimentally determined protein–peptide complexes based on the following characteristics.^31,32^ The peptide sequence lengths were set to 5 to 30 amino acids, and the refinement resolution was set to a maximum of 2 Å. To keep consistency, only structures that were compatible with the PDB format were selected. To ensure that none of the complexes were present in the training sets of any of the three co-folding methods, a release date cut-off of after 01 June 2023 was applied, as Boltz-2 includes complexes up to 01 June 2023 in its training data. A protein mutation count of 0 was applied.

In total, 715 PDB entries were extracted from the RCSB PDB. TMalign was applied to cluster the protein–peptide complexes based on structural similarity. A stringent TM-score threshold of 0.9 was chosen to decrease the risk of redundant structure complexes. The TM-score was calculated globally for the structures contained in the PDB files and was not restricted to the peptide-binding interface. Consequently, complexes with highly similar protein folds but distinct peptide-binding modes or interfaces may have been clustered together, making this filtering approach more likely to exclude distinct interfaces than to retain structurally redundant complexes. For every cluster, a representative score was assigned based on resolution, atom count (higher values favored to reduce the risk of truncated structures), and experimental method, with X-ray diffraction as the preferred method, followed by electron microscopy, and lastly NMR. The structures with the highest representative score were included to construct the independent dataset. The remaining complexes were investigated to determine whether the peptides contained any missing amino acids within the chain and every complex contained a peptide of 5–30 amino acid length. The final independent dataset contained 57 structurally distinct protein–peptide complexes, for which a detailed table is provided in the SI.

Receptor-level structural redundancy with the trainingset was assessed by extracting the annotated receptor chains from each dataset structure and searching them against the Protein Data Bank using Foldseek (v10).^33^ Only candidate structures released before 1 June 2023 were retained. For each dataset receptor chain, the Foldseek candidates were ranked by structural similarity, and up to the 500 highest-ranking pre-cutoff candidates were evaluated using TM-align. For each receptor pair, the maximum reported TM-score, obtained from the scores normalized by the lengths of the dataset and candidate receptor chains, was used as the structural-similarity measure. Complex-level structural redundancy was defined as the presence of at least one pre-cutoff structure with a maximum TM-score (TM_max_) of ≥0.90.

Peptide-interface redundancy was subsequently evaluated only for complex pairs that passed the threshold of TM_max_ ≥ 0.90. For each dataset complex, up to the 500 highest-scoring pre-cutoff TM-align hits were retained for this analysis. Within each retained candidate structure, all non-receptor protein chains containing 3–50 residues were considered potential peptide chains. Receptor-peptide interface residues were defined as receptor residues containing at least one heavy atom within 5.0 Å of a peptide heavy atom. The candidate receptor sequence was globally aligned to the corresponding dataset receptor sequence to map candidate interface residues onto the dataset receptor. Peptide sequence identity was calculated as the number of identical aligned residues divided by the length of the longer peptide sequence. Interface overlap was calculated as the fraction of receptor-interface residues in the dataset complex that were also identified as interface residues in the mapped candidate complex. Potential complex-level redundancy relative to the method's training sets was classified into four categories: high complex-level redundancy, moderate complex-level redundancy, peptide-level non-redundancy, and complex-level non-redundancy.

Moderate complex-level redundancy was defined as a receptor TM_max_ > 90, a peptide sequence identity ≥40%, and an interface overlap ≥50%. High complex-level redundancy required a receptor TM_max_ > 90, a peptide sequence identity ≥80%, and an interface overlap ≥50%. Peptide-level non-redundancy was defined as a receptor TM_max_ > 90 combined with either a peptide sequence identity <40% or an interface overlap <50%. Complex-level non-redundancy was defined as a receptor TM_max_ ≤ 90.

### Dataset structure characterization and annotation

The two datasets of protein structures were combined and analyzed with a phylogenetic analysis. The phylogenetic analysis was performed using IQ-TREE (v3.0.1).^34^ The MSA was generated with Clustal Omega (v1.2.4), and the optimal amino acid substitution model was determined by ModelFinder.^35,36^ A maximum-likelihood tree was created with sequence similarity distances (branch lengths) measured in substitutions per site. For example, when two protein sequences have a branch length of 2 between each other, each aligned amino acid position has undergone two substitutions on average during evolutionary divergence, indicating evolutionary distance. Statistical branch support was evaluated using both SH-aLRT (1000 replicates) and ultrafast bootstrap (1000 replicates).^37,38^ The resulting tree was annotated and visualized using the free access mode of the web-based Interactive Tree of Life (iTOL, v1.0) platform.^39^

### Docking with AutoDock-CrankPep and HADDOCK

Two established protein–peptide docking platforms were selected for comparative analysis: AutoDock-CrankPep (ADCP, v1.1), and HADDOCK (webserver, v2.5-2024.12).^25,27^ The features of ADCP and HADDOCK are summarized in Table 1, and the key parameters are listed below.

**Table 1:** Overview of benchmarked methods

Method	Type^a^	Input^b^	Sampling algorithm	Scoring function^c^
AutoDock-CrankPep	PpD	cR, cP or sP, BS^d^	CRANKITE Metroplolis Monte Carlo sampling	Gō-type potential, Ramachandran propensities, AutoDock affinity grids
HADDOCK	PpD	cR, cP, AIR^d^	Randomized search algorithm	Weighted combination of vdW, electrostatics, desolvation, AIR restraints, and BSA
AlphaFold-Multimer	CF	sR, sP, BS^d^	Invariant point attention (IPA)-based neural network. End to end structure prediction	Learned probabilistic confidence metrics. Global confidence *via* ipTM + pTM
AlphaFold3	CF	sR, sP, BS^d^	Diffusion-based generative structure prediction with 4 PairFormer layers	Learned probabilistic confidence metrics. Global confidence *via* ipTM, pTM, and clashes + disorder compensation
Boltz-2	CF	cR, sP, BS^d^	Diffusion-based generative structure prediction with 8 PairFormer layers and Kabsch diffusion interpolation	Learned probabilistic confidence metrics. Global confidence *via* ipTM and pLDDT

a PpD: protein–peptide docking. CF: co-folding.

b cR: receptor conformation. cP: peptide conformation. sR: receptor sequence. sP: peptide sequence. BS: binding site. AIR: ambiguous interaction restraints.

c vdW: van der Waals interaction energy. BSA: buried surface area. pTM: predicted template modelling score. ipTM: interface predicted template modelling score. pLDDT: predicted local distance difference test.

d Optional parameters.

#### ADCP

ADCP docking was performed on an Intel Xeon Gold 6126 CPU. For each ADCP docking run, the number of replicas evaluated was set to 200. All peptides were docked with 30 million Monte-Carlo (MC) steps per replica. The number of MC steps is based on the principle of 1 million MC steps per amino acid docked, as proposed by the ADCP documentation.^40^ As the peptides within the benchmark contain an amino acid length range of 5 to 30, and to keep dock parameters consistent, 30 million MC steps were chosen across the whole dataset. In this study, we applied a site-guided docking scenario in which the approximate binding region was known, while the peptide conformation, orientation, and specific interaction pattern remained unknown. This setting was selected to enable a more balanced comparison between molecular docking and co-folding methods. For site-guided docking, an approximate binding location was defined using a grid box size based on the peptide length, as proposed by Weng *et al.*^28^ The length of the three sides of the grid box was set to (peptide amino acid length ×3.8) Å, with the grid box centered around the position of the reference peptide in relation to the unbound protein structure. The value of 3.8 represents the distance between two adjacent Cα atoms. OpenMM, an open-source toolkit for molecular dynamics simulations, was used to perform minimization steps after docking followed by reranking. This generated a new score which is called the OMM score. The top-20 docking results are reranked with “sannerlab” OpenMM parameters based on the amber99sb forcefield. Reranking is performed based on interaction energy (*E*_complex_ − *E*_receptor_ − *E*_peptide_). Minimization is done with 1000 steps in an implicit solvent, as no explicit solvent option was available. A seed of 42 was chosen for reproducibility. For complex 8WXW, two domains corresponding to amino acid residues 403–699 and 721–966 were removed prior to ADCP docking to reduce the size of the receptor structure and enable successful execution of the docking calculations.

#### HADDOCK

HADDOCK docking was performed with expert mode of the HADDOCK server.^27^ The default settings provided by the web server were used, unless specified below. The unbound protein structures were used as protein structure input. The target residues that were located within 5 Å of the peptide residues in the bound protein–peptide complex were retrieved with an in-house script and were set as ‘active’ residues on the unbound protein. ‘Passive’ residues were automatically defined around the active residues. The ‘active’ and ‘passive’ residues help HADDOCK score the interactions and poses of the peptides, favoring interactions with ‘active’ residues and accepting passive residues while punishing interactions with ‘inactive’ residues. The peptide residues were all defined as ‘passive’. Peptides were docked fully flexible. Rigid body docking, semiflexible refinement, and solvent refinement were set to 6000, 400, and 400, respectively. Short solvent refinement molecular dynamics was done with explicit solvent. Clustering was done by RMSD, with a cutoff value of 5 Å. The Minimum cluster size was set to 4.

### Co-folding with deep learning methods

Three deep learning-based co-folding methods were included for comparative analysis to assess their capability in predicting protein–peptide complex structures, namely AFM (v2.3.2), AF3 (v3.0.1), and Boltz-2 (v2.1.1), which are summarized in Table 1 and the key parameters are listed below.

#### AlphaFold-Multimer

AFM predictions were performed using NVIDIA GeForce GTX 1080 GPUs in multimer mode. From the unbound protein PDB files, the sequences were extracted and used as protein input. The peptide sequences were extracted from the peptide PDB files and used as binder input. The maximum template date was set to 2022-01-01. For each entry, 25 structures were generated. The predicted local distance difference test (pLDDT) scores from the peptides were extracted (peptide pLDDT). By default, AFM uses the ipTM + pTM values for ranking the complexes. Furthermore, the ipTM + pTM, ipTM, and peptide pLDDT were extracted.

#### AlphaFold3

AF3 predictions were conducted on an NVIDIA A100 GPU using 10 recycling steps and 25 diffusion samples, consistent with AlphaFold's default settings. A maximum template date of 01-06-2023 was applied. The databases uniprot_all_2021_04, uniref. 90_2022_05, and mgy_clusters_2022_05 were used for sequence alignment searches. Default settings were used for the remaining parameters. By default, AF3 uses the ranking score values for ranking the complexes. In addition, the ranking score, ipTM, and peptide pLDDT were extracted.

#### Boltz-2

Boltz-2 entries were conducted on an NVIDIA A100 GPU using 10 recycling steps and 25 diffusion samples to ensure a fair comparison with AlphaFold. A maximum template date of 01-06-2023 was applied. Inference-time potentials were included to guide the model toward more physically realistic structures. The MMseqs2 server was used for automatic MSA generation.^41^ A greedy MSA pairing approach was chosen. Default settings were used for the remaining parameters. By default, Boltz-2 uses the confidence score (confidence score = 0.8 × overall pLDDT + 0.2 × interface pTM) values for ranking the complexes. The ranking score, ipTM, peptide pLDDT, complex iPDE, and complex ipLDDT were extracted.

### Pose prediction performance analysis of methods

The methods were benchmarked based on the pose quality calculated with DockQ (v2).^42^ DockQ is measured using a combination of fraction of native contacts (Fnat), ligand root mean squared deviation (LRMSD), and interface root mean squared deviation (iRMSD) to score the predicted pose based on the experimental pose from 0 to 1, with 1 being a perfect match. Classification of pose quality was determined in agreement with CAPRI guidelines and in accordance with DockQ documentation.^43,44^ Poses were classified with high, medium, or acceptable accuracy corresponding with a DockQ ≥ 0.80, 0.49 ≤ DockQ < 0.80, and 0.23 ≤ DockQ < 0.49, respectively. A DockQ ≤ 0.22 was considered incorrect.

### Assessment of ranking accuracy and early recognition performance

To assess monotonic agreement between predicted and experimental values, an average Spearman's rank correlation coefficient (*ρ*) and an average Kendall's *τ* were calculated. Global discriminative ability was quantified using the area under the receiver operating characteristic curve (ROC-AUC), computed using a rank-based Mann–Whitney formulation.^45^ The average precision (PR-AUC) was used to evaluate enrichment of good poses within the top-ranked predictions, providing sensitivity to class imbalance.^46,47^

To characterize early recognition performance, a Boltzmann-Enhanced Discrimination of ROC (BEDROC) was calculated using an exponential weighting parameter (*α*) of 20, as proposed by Truchon *et al.*, to quantify how effectively positives appear at the highest-ranked positions.^48^ With *α* = 20, 80% of the maximum contribution to the BEDROC comes from the first 8% of the ranked list.

To estimate uncertainty, non-parametric bootstrap resampling was performed with 10 000 replicates. In each replicate, the dataset was sampled with replacement and all metrics were recalculated. The 95% confidence intervals (CIs) were derived from the empirical percentile method (2.5th and 97.5th percentiles of the bootstrap distribution).^49^ All metrics were computed using in-house R scripts (R v4.4.1) to ensure reproducibility and consistent handling of ranking information across score types.

## Results

### Most complexes in the independent test set show low or no redundancy with the training set

To evaluate whether the independent test set contained structural similarity to the co-folding trainings on protein-, peptide-, and complex-level, a structural redundancy analysis was performed for all 57 complexes. Eleven receptors had no pre-cutoff training-set structure with a TM_max_ ≥ 0.90 and were therefore classified as structurally nonredundant. The remaining 47 receptors showed high structural similarity to at least one training-set receptor and were subsequently assessed for peptide-sequence and receptor-peptide interface redundancy. Of these, 27 complexes showed low peptide-interface leakage, based on low peptide sequence identity and/or limited interface overlap with the corresponding training-set structures. Eighteen complexes were classified as having moderate complex-level leakage, whereas two met the criteria for high complex-level leakage. Overall, 37 of the 57 complexes were either structurally nonredundant or showed low peptide-interface leakage relative to the training set.

### Benchmark datasets contained primarily evolutionarily distant proteins for pose prediction performance analysis

**Fig. 2:**
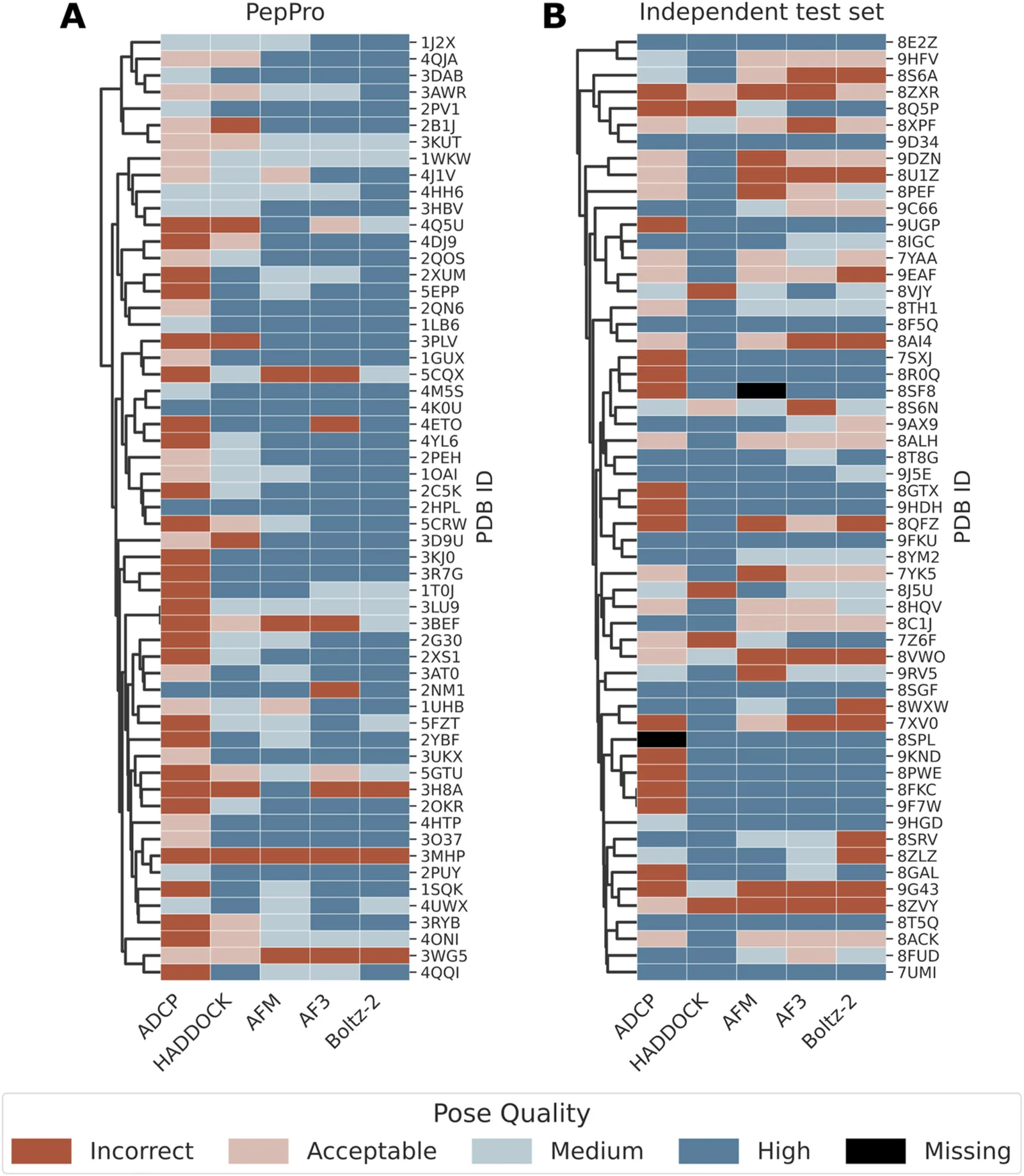
Pose prediction performance of the top-10 predictions per method, clustered based on evolutionary sequence similarity. Success rate of AutoDock-CrankPep (ADCP), HADDOCK, AlphaFold-Multimer (AFM), AlphaFold3 (AF3), and Boltz-2 on (A) the PepPro dataset and (B) the independent test set. Success rate is defined as the percentage of complexes achieving a given pose quality classification relative to all classified complexes. Pose quality was assessed using the DockQ score, with categories defined as high (≥0.80), medium (0.49–0.79), acceptable (0.23–0.48), and incorrect (≤0.22).

To assess whether pose prediction performance is influenced by evolutionary relatedness and sequence similarity among protein targets within the datasets, we first analysed protein similarity in both the benchmark and independent datasets. Pairwise sequence similarity distances, expressed as substitutions per site, were used to cluster all protein–peptide complexes as shown in Fig. 2. The maximum likelihood phylogenetic tree are provided in the SI (Fig. S1). Across both datasets, most proteins exhibited substantial evolutionary divergence (>1 substitution per site). PDB entries with a sequence distance lower than 1 substitution per site typically corresponded to identical proteins with close to 0 rather than closely related homologs.

In the PepPro dataset, only a single case of identical proteins was observed. PDB entries 3BEF and 3LU9 both contain prothrombin, with a sequence similarity distance of approximately 0. In the independent dataset, a comparable case was identified for PDB entries 8FKC and 9F7W, which both correspond to peroxisome proliferator-activated receptor gamma (PPARG) and are therefore evolutionarily closely related.

Across the combined benchmark datasets, additional identical protein pairs were identified. Specifically, 5GTU and 8FUD both contain vacuolar protein sorting-associated protein (Vps), and 3UKX and 7UMI both contain importin subunit alpha-1, each with sequence similarity distances close to 0. The remaining proteins across both datasets were widely diverse and non-similar, with sequence similarity distances typically exceeding 1 substitution per site.

The PDB entries with similar proteins were evaluated based on pose prediction performance to determine whether sequence similarity of the protein influenced model behaviour. The DockQ score was used to classify predictions into incorrect, acceptable, medium, and high quality.

The peptides in PDB entries 3BEF and 3LU9, both targeting prothrombin, were highly similar. The 9-mer peptide in 3BEF represents a subset of the 25-mer peptide in 3LU9. However, the peptides bind to distinct motifs on prothrombin. As shown in Fig. 2A, Boltz-2 achieved medium-quality pose predictions for both complexes, correctly identifying two separate binding motifs and assigning each peptide to the appropriate site. In contrast, AFM and AF3 only correctly predicted the binding pose for the 3LU9 peptide.

The peptides in PDB entries 5GTU and 8FUD (Vps), as well as 3UKX and 7UMI (importin subunit alpha-1), differ substantially in sequence and length, sharing no detectable similarity. However, the binding motifs on the proteins are comparable across complexes, meaning that binding site prioritization was not required during co-folding. As illustrated in Fig. 2, pose prediction performance was consistent across these systems and did not appear to be affected by either peptide dissimilarity or training set availability.

We subsequently evaluated pose prediction performance on both datasets to identify similar performance patches. Fig. 2B showed a clear patch of complexes (8SPL, 9KND, 8PWE, 8FKC, 9F7W, and 9HGD) for which all methods could predict a high-quality pose. When assessed based on sequence similarity distance, only 8FKC and 9F7W were evolutionarily related (both PPARG proteins). The remaining proteins were widely diverse and non-similar, with the closest sequence similarity distance observed between 8PWE and 8FKC (1.41 substitutions per site). No consistent relationship between evolutionary distance and pose prediction success was observed in the benchmark datasets. The built-in minimization step of ADCP failed for the 1 T0J and 3RYB complexes; therefore, the ADCP score was used instead of the OMM score to rank the predicted poses. For 8SPL, ADCP failed to reduce the protein structure, preventing docking from being performed. For 8SF8, AFM repeatedly stopped because of an HH-suite MSA-generation failure, resulting in an unsuccessful prediction run.

### Co-folding methods outperform molecular docking methods on conformational flexible proteins

The pose prediction performance of two molecular docking methods and three co-folding methods were assessed on the PepPro database to determine the methods' capabilities to predict poses on difficult, conformationally flexible protein targets. The DockQ score was used to classify predictions into incorrect, acceptable, medium, and high quality across the top 1, 3, 5, 10, and 20 ranks. The detailed table of quality classification percentages for the separate top ranks on the PepPro dataset can be found in the SI (Table S2).

**Fig. 3:**
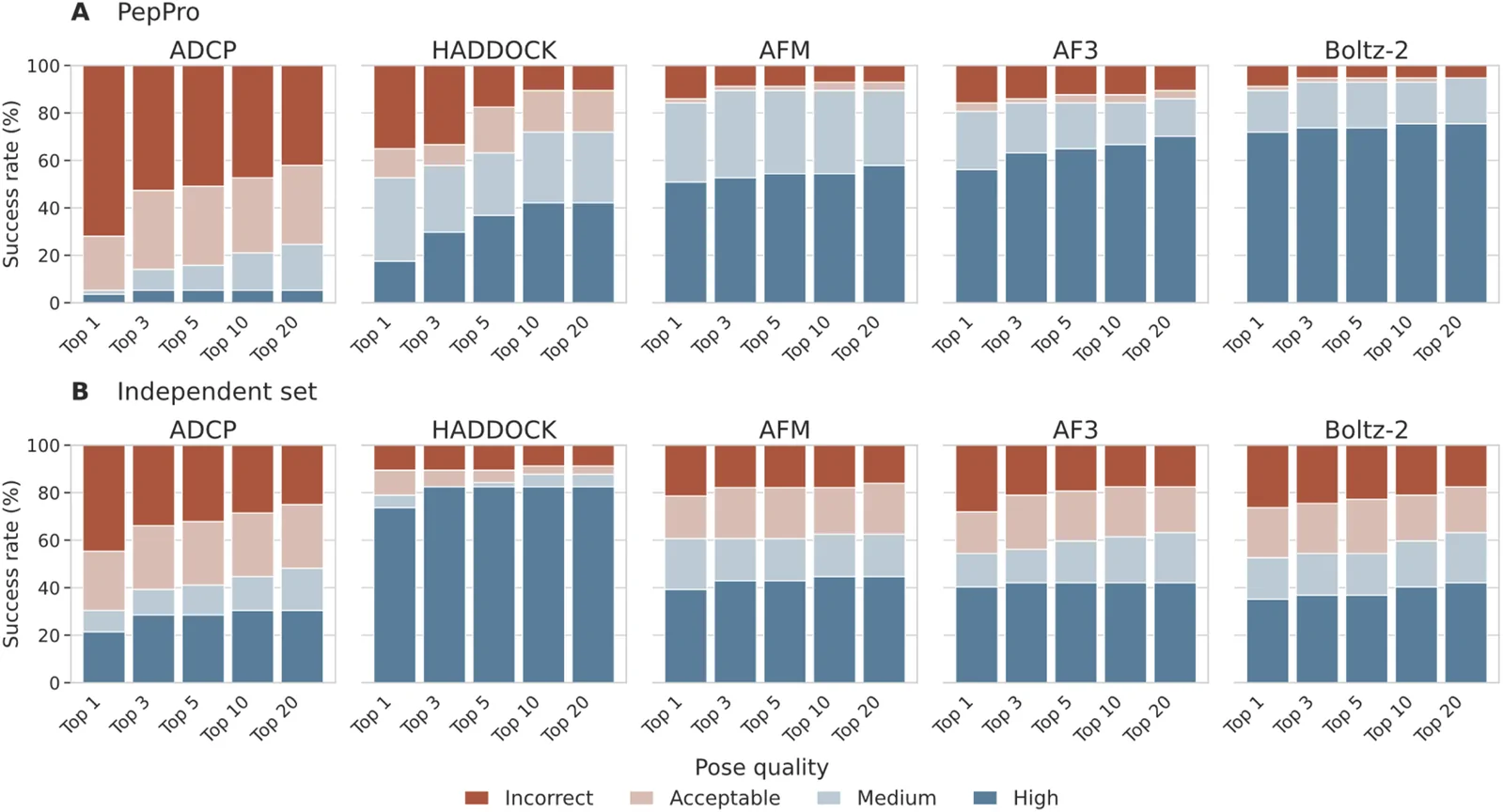
Success rate of peptide pose prediction by molecular docking and co-folding methods. Success rate of AutoDock-CrankPep (ADCP), HADDOCK, AlphaFold-Multimer (AFM), AlphaFold3 (AF3), and Boltz-2 on (A) the PepPro dataset and (B) the independent test set. Success rate is defined as the percentage of complexes achieving a given pose quality classification relative to all classified complexes. Pose quality was assessed using the DockQ score, with categories defined as high (≥0.80), medium (0.49–0.79), acceptable (0.23–0.48), and incorrect (≤0.22). Success rates are reported across the top 1, 3, 5, 10, and 20 ranked poses.

Compared to the co-folding methods, the two molecular docking methods, ADCP and HADDOCK, obtained a lower success rate of correctly posed peptides. Especially ADCP performed drastically worse than the other methods in this setting, as shown below in Fig. 3A. ADCP produced a large proportion of incorrect top-ranked predictions, reaching 73.6%, but showed a gradual increase in acceptable predictions or higher, reaching up to 58.5% in the top-20 set. However, medium-quality docking poses remained limited at ≤13.2%, and high-quality docking poses appeared only sporadically, never exceeding 5.7%.

HADDOCK showed a lower rate of incorrect predictions and demonstrated a greater ability to generate higher-quality models than ADCP, reaching a high-quality pose for 43.6% of the protein–peptide complexes in the top-10 set, as shown in Fig. 3A. Furthermore, within the top-5 set prediction, more than 50% of the predicted poses were of medium-quality or higher, and 80% of the predictions had an acceptable-quality or higher. The percentage of high-quality predictions did not increase in the top-20 set compared to the top-10 set, and only marginally increased in the top-10 set compared to the top-5 set, suggesting that correct HADDOCK pose predictions are ranked at the top.

AFM generated a substantial proportion of high-quality predictions, achieving 50.9% in the top-1 ranked predictions, increasing up to 57.9% in the top-20 predictions. Incorrect predictions were generally low, ranging from 14.0% (top-1) to 7.0% (top-10 and top-20). AF3 showed similar trends, with high-quality predictions peaking at 68.4% in the top-20 set. Incorrect predictions decreased as more predictions were considered, while acceptable and medium-quality predictions remained relatively stable across ranks. Boltz-2 achieved the highest proportion of high-quality models among the examined methods, generating a high-quality pose in the top-ranked prediction 68.4% of the time and reaching the top performance of 73.7% in the top-20 predictions. For all co-folding methods, the proportion of high-quality models increased only marginally across the ranking depth. The largest gain observed was for AF3, which showed a 12.0% increase from the top-1 to the top-20 predictions. This limited growth indicates that these models already capture most of their predictive power in the top-ranked pose.

**Fig. 4:**
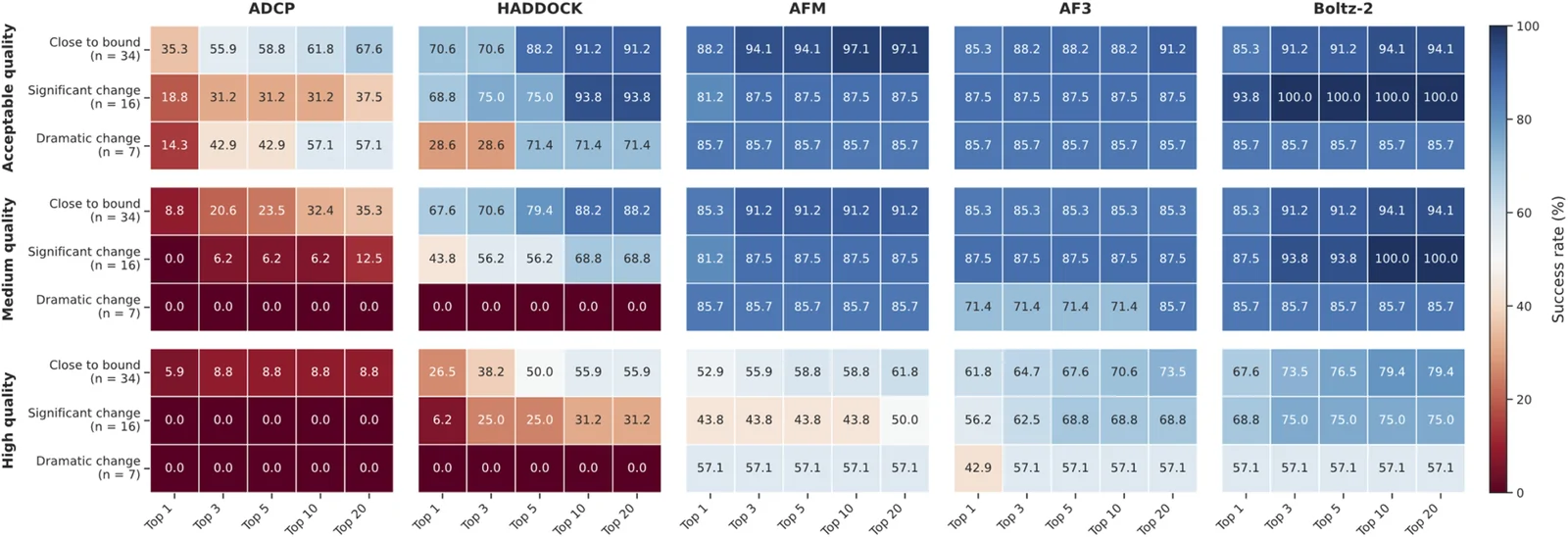
Success rates of protein–peptide pose-prediction methods across conformational-change categories. Heatmaps show the percentage of protein–peptide complexes for which each docking method generated at least one acceptable-, medium-, or high-quality pose within the indicated top-ranked predictions. Rows correspond to conformational-change categories based on the backbone interface root-mean-square deviation (I-RMSD_b) of the unbound structure relative to the bound structure: close to bound (I-RMSD_b < 1.0 Å), significant conformational change (1.0 ≤ I-RMSD_b < 2.0 Å), and dramatic conformational change (I-RMSD_b ≥ 2.0 Å). The number of complexes in each category is shown in parentheses. Columns indicate the top (N) poses used to evaluate docking success, and values within the cells represent success rates as percentages. The same colour scale, ranging from 0% to 100%, is used across all docking methods and quality thresholds to facilitate direct comparison. Complexes for which a method did not generate a prediction were retained in the denominator and counted as unsuccessful.

Fig. 4 shows that the co-folding methods generally achieved higher success rates than the molecular docking methods across the different complex-quality and conformational-change categories. AFM, AF3, and Boltz-2 showed consistently high performance for acceptable- and medium-quality complexes, with success rates frequently exceeding 85% from the top-ranked prediction onward. In comparison, the docking methods showed a stronger dependence on both the quality of the experimental complex and the number of predictions considered.

Among the molecular docking methods, HADDOCK performed substantially better than ADCP and approached the performance of the co-folding methods in several categories. For acceptable-quality complexes with a close-to-bound conformation, HADDOCK increased from 70.6% for the top-1 prediction to 91.2% for the top-10 and top-20 predictions. This final success rate was comparable to AF3, which reached 91.2%, and only slightly lower than AFM and Boltz-2, which reached 97.1% and 94.1%, respectively. A similar pattern was observed for acceptable-quality complexes with a significant conformational change. HADDOCK reached 93.8% at top-10, exceeding the 87.5% success rates of AFM and AF3, although Boltz-2 achieved 100%. For acceptable-quality complexes with a dramatic conformational change, HADDOCK improved from 28.6% at top-1 to 71.4% at top-5, whereas the co-folding methods consistently reached 85.7%.

The co-folding methods remained more consistent in obtaining medium-quality predictions for complexes within the significant and dramatic conformational change classifications. For significant conformational changes, HADDOCK reached 68.8% at top-10, compared with 87.5% for AFM and AF3 and 100% for Boltz-2. No successful medium-quality models were obtained by ADCP and HADDOCK for complexes with a dramatic conformational change, whereas the co-folding methods maintained success rates between 71.4% and 85.7%.

The largest differences between docking and co-folding were observed for high-quality complexes. ADCP produced success rates below 10% in most high-quality categories and did not generate successful models for complexes with significant or dramatic apo-to-holo structural differences. HADDOCK showed a gradual improvement for close-to-bound complexes, from 26.5% at top-1 to 55.9% at top-10, but remained below the co-folding methods, which reached 61.8–79.4% at top-20. For complexes with significant conformational changes, HADDOCK increased from 6.2% to 31.2%, whereas AFM, AF3, and Boltz-2 reached 50.0%, 68.8%, and 75.0%, respectively. Neither docking method generated high-quality models for complexes with dramatic conformational changes, while all three co-folding methods achieved success rates of approximately 57.1% at top-3 or higher.

Overall, the co-folding methods showed consistent and comparable performance across the evaluated categories. Nevertheless, their success rates also decreased as the structural difference between the docked apo conformation and the holo conformation increased, particularly for high-quality models. For acceptable- and medium-quality complexes, performance remained relatively stable, although success rates for complexes with dramatic conformational changes were generally lower than for the close-to-bound and significant categories. Among the complexes for which two or more co-folding methods failed to identify a correct pose, nearly all showed substantial conformational differences between their apo and holo protein structures. The 3MHP complex was an exception, as it did not exhibit a similarly pronounced conformational change. The reason for the consistent prediction failure of this complex remains unclear.

### Co-folding pose quality prediction decreases substantially when benchmarked on peptide- and complex-level non-redundant complexes

Due to the tendency of the co-folding methods to overfit poses of proteins based on an internal training set and to investigate the generalization capability, the performance of the co-folding methods was benchmarked on the curated independent dataset, which incorporates protein–peptide complexes that have some form of non-redundancy with their respective training sets. The DockQ score was used to classify predictions into Incorrect, acceptable, medium, and high quality across the top 1, 3, 5, 10, and 20 ranks. The percentages of quality classifications for the separate top ranks on the independent test set can be found in the SI (Table S3).

As shown in Fig. 3B, the molecular docking methods, especially HADDOCK, performed substantially better on the independent dataset compared to the PepPro dataset. Without the impact of apo–holo conformational change, HADDOCK substantially outperformed all the other methods. HADDOCK achieved a high-quality pose-prediction success rate of 73.7% for the top-1 prediction, increasing to a maximum success rate of 82.5% when the top three predictions were considered. For only 5.3% of the complexes, the method failed to generate an acceptable pose.

**Fig. 5:**
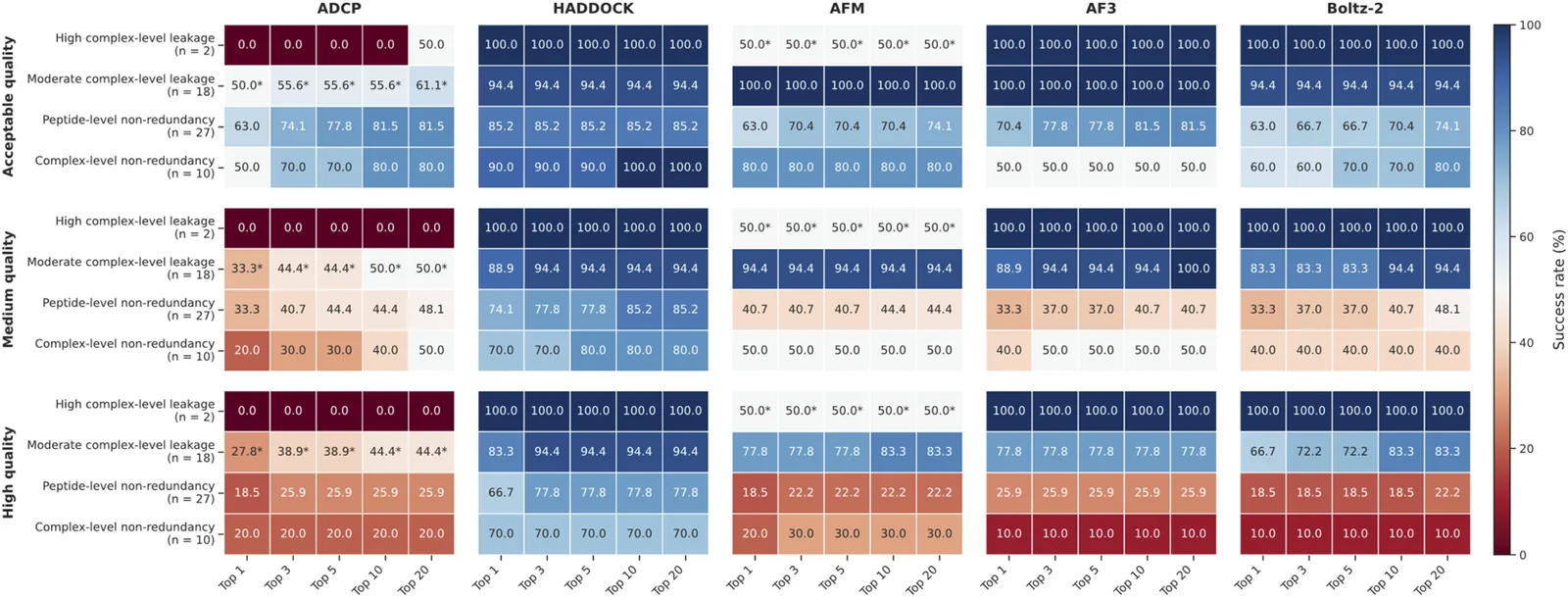
Success rates of peptide–protein docking methods across data-leakage categories. Heatmaps show the percentage of protein–peptide complexes for which each docking method generated at least one acceptable-, medium-, or high-quality pose within the indicated top-ranked predictions. Rows correspond to data-leakage classifications, with the number of complexes in each category shown in parentheses. Columns indicate the top N poses used to evaluate docking success. Values within the cells represent success rates as percentages. The same colour scale, ranging from 0% to 100%, is used across all methods and quality thresholds to facilitate direct comparison. Complexes without a corresponding prediction were included in the denominator and counted as unsuccessful. *The method failed to generate a prediction for one or more complexes within this classification.

All co-folding methods performed substantially worse on the independent dataset than on the PepPro dataset. As shown in Fig. 5, the high performance observed on PepPro was strongly associated with the presence of peptide-related information in the co-folding training datasets, suggesting that data leakage contributed to the reported results. When overlapping or related peptide data were available, all the co-folding methods achieved near-perfect performance, with medium-quality pose-prediction success rates of approximately 94.4–100%. In contrast, performance decreased substantially when peptide-related information was excluded. The success rate for medium-quality pose predictions dropped to 33.3–48.1% and for high-quality pose predictions the success rate dropped to 10–30% when some kind of non-redundancy is present. AFM achieved a pose-prediction success rate of 50% in the high complex-level redundancy category. However, this result should be interpreted cautiously because the category contained only two complexes. Across the non-redundant subsets, AFM generally achieved slightly higher success rates than the other methods, whereas Boltz-2 tended to perform slightly worse. This trend was most pronounced for high-quality predictions on the complex-level non-redundant subset. Here, AFM reached a success rate of 30%, compared with 10% for both AF3 and Boltz-2. As this subset contained ten complexes, AFM successfully predicted three complexes, corresponding to two additional successful predictions relative to AF3 and Boltz-2. Overall, these results indicate that AFM showed the strongest generalization to non-redundant complexes, with AF3 very close behind and Boltz-2 generally showed the weakest performance.

When looking at the overall independent test set pose prediction performance in Fig. 3B, AFM demonstrated a performance of 39.3% high-quality top-1 predictions and reaching 44.6% high-quality predictions in the top-20 set. Although its overall performance was lower than on the PepPro benchmark, the reduction in high-quality models was less pronounced compared to the other methods. As a result, AFM outperforms, however slightly, both AF3 and Boltz-2. Fig. 5 showed that AFM indeed managed to generate more high-quality pose prediction for complex-level non-redundant structures, obtaining a 30.0% pose prediction success rate compared to AF3's and Boltz-2's 10%.

AF3 showed a similar pattern, generating 40.4% high-quality predictions at the top rank and increasing to 42.1% in the top-20 set. Compared to the PepPro dataset, AF3 exhibited a substantial decrease in high-quality predictions, suggesting that its prior performance was highly influenced by training set similarity. However, the performance is still comparable to the other 2 methods on the independent data set.

Boltz-2 displayed the highest performance drop of the three methods compared with the PepPro dataset. High-quality predictions were limited to 35.1% at the top rank, increasing to 42.1% in the top-20 set. While Boltz-2 previously provided the strongest performance, its accuracy decreases on the independent dataset, indicating a stronger dependence on training-set familiarity compared to the other methods.

### Co-folding obtained better ranking performance then molecular docking across all methods and metrics

To examine the relationship between structural pose prediction performance and individual scoring metrics, the methods were evaluated based on their scoring and ranking performance on the PepPro dataset to assess their ability to capture high conformational variability. Moreover, since the protein–peptide complexes in the PepPro dataset are included in the co-folding methods' training sets, this evaluation also serves as an internal validation of those methods. The performance was measured using rank-based correlation metrics (Spearman's *ρ*, Kendall's *τ*), classification-oriented metrics (ROC-AUC, PR-AUC), and early recognition (BEDROC). The detailed results are listed in Table 2. A point plot visualizing the performance of the scoring methods on the PepPro dataset is provided in the SI (Fig. S2).

**Table 2:** Assessment of ranking correlation (Spearman *ρ*; Kendall *τ*), ranking accuracy (ROC-AUC; PR-AUC), and early recognition performance (BEDROC, *α* = 20) for two molecular docking methods and three co-folding methods on the PepPro dataset. All metrics were evaluated using a bootstrapped mean and a 95% confidence interval

Method metric	Mean Spearman *ρ*	Mean Kendall *τ*	Mean ROC-AUC	Mean PR-AUC	Mean BEDROC
**AutoDock-CrankPep**					
ADCP score	−0.58 ± 0.04	−0.39 ± 0.03	0.46 ± 0.21	0.01 ± 0.0	0.00 ± 0.0
OMM score	−0.40 ± 0.05	−0.27 ± 0.03	0.59 ± 0.22	0.01 ± 0.01	0.00 ± 0.0

**HADDOCK**					
HADDOCK score	−0.09 ± 0.05	−0.06 ± 0.03	**0.82** ± **0.04**	0.15 ± 0.08	0.21 ± 0.09

**AlphaFold-Multimer**					
ipTM	0.67 ± 0.03	0.48 ± 0.02	0.79 ± 0.02	0.69 ± 0.04	0.80 ± 0.06
ipTM + pTM	0.66 ± 0.03	0.47 ± 0.02	0.78 ± 0.02	0.67 ± 0.04	0.77 ± 0.06
Peptide pLDDT	**0.73** ± **0.03**	**0.53** ± **0.03**	0.80 ± 0.02	0.71 ± 0.04	0.83 ± 0.05

**AlphaFold3**					
ipTM	0.54 ± 0.04	0.38 ± 0.03	0.76 ± 0.03	0.74 ± 0.04	0.74 ± 0.08
Ranking score	0.50 ± 0.04	0.34 ± 0.03	0.71 ± 0.03	0.68 ± 0.04	0.62 ± 0.08
Peptide pLDDT	0.53 ± 0.04	0.36 ± 0.03	0.74 ± 0.03	0.74 ± 0.03	0.83 ± 0.05

**Boltz-2**					
ipTM	0.44 ± 0.05	0.30 ± 0.04	0.77 ± 0.03	0.84 ± 0.03	0.89 ± 0.04
Confidence score	0.64 ± 0.03	0.46 ± 0.03	**0.85** ± **0.02**	**0.91** ± **0.02**	**0.98** ± **0.01**
Peptide pLDDT	0.56 ± 0.04	0.40 ± 0.03	0.79 ± 0.03	0.85 ± 0.03	0.89 ± 0.04
Complex iPDE	0.53 ± 0.04	0.38 ± 0.03	0.76 ± 0.03	0.86 ± 0.02	0.93 ± 0.03
Complex ipLDDT	0.65 ± 0.03	0.46 ± 0.03	**0.83** ± **0.02**	**0.92** ± **0.01**	**0.99** ± **0.01**

ADCP performed generally worse than the other methods on the PepPro dataset. The ADCP score and OMM score showed poor performance with low confidence across all evaluation criteria. Both metrics exhibited negative rank correlations with experimental data (mean Spearman *ρ* down to −0.58 ± 0.04; Kendall *τ* down to −0.39 ± 0.03), low discriminative ability (ROC-AUC ≤0.59 ± 0.22), negligible mean precision-recall performance (PR-AUC ≈ 0.01 ± 0.01), and no early enrichment (BEDROC = 0.00). The large variability further highlights the instability and limited predictive value of ADCP.

HADDOCK showed greatly improved performance in PR-AUC, ROC-AUC, and BEDROC compared to ADCP, but performed considerably worse than the co-folding methods on correlation and ranking performance. The HADDOCK score showed a negligible inverse ranking correlation (Spearman *ρ* = −0.09 ± 0.05) but achieved strong discriminatory performance (ROC-AUC = 0.82 ± 0.04), ranking among the best-performing scores across all methods. Early enrichment (BEDROC = 0.21 ± 0.09) is present, but low. This indicated that, despite limited ranking confidence, the HADDOCK score can moderately separate high-from low-quality complexes.

When evaluating the co-folding methods on the PepPro dataset, the correlation and ranking performance increased drastically. For AFM, the ipTM, ipTM + pTM, and peptide pLDDT metrics demonstrated strong and reproducible performance with comparatively low variance. ipTM and ipTM + pTM achieved moderate correlations (Spearman *ρ* ≈ 0.66–0.67) and good discriminative power (ROC-AUC ≈ 0.78–0.79). Peptide pLDDT showed the highest ranking correlation with the PepPro dataset compared to all metrics from all methods (Spearman *ρ* = 0.73 ± 0.03; Kendall *τ* = 0.53 ± 0.03). Furthermore showed a robust ROC-AUC (0.80 ± 0.02), a moderately high PR-AUC (0.71 ± 0.04), and strong early enrichment (BEDROC = 0.83 ± 0.05).

AF3 performed closely similar to AFM and Boltz-2, but never outperformed them on either correlation or ranking performance when conformationally flexible protein–peptide complexes were considered. The Ranking Score, ipTM score, and peptide pLDDT showed moderate to low ranking correlation performance, with Spearman correlations around 0.50–0.54 and Kendall *τ* correlation around 0.34–0.38. ROC-AUC values between 0.71 and 0.76 ± 0.08 showed moderate classification performance. The peptide pLDDT showed the best early enrichment (BEDROC = 0.83 ± 0.05) within AF3, supporting reliable prioritization of top candidates.

As reflected by the pose prediction performance, Boltz-2 outperformed the other methods on ranking and early-enrichment performance on the PepPro dataset. The complex ipLDDT, and complex pLDDT achieved the highest discriminative power (ROC-AUC up to 0.85 ± 0.02), highest precision-recall performance (PR-AUC up to 0.92 ± 0.01), and a near-perfect early enrichment (BEDROC up to 0.99 ± 0.01) compared to the other methods on the PepPro dataset. The correlation of complex ipLDDT, and complex pLDDT with experimental values were moderate (Spearman's *ρ* = 0.64–0.65; Kendall's *τ* = 0.46–0.47), not surpassing the correlation performance of AFM.

### AlphaFold's architecture seems to perform better on the independent dataset, while Boltz-2's performance decreases

The performance of the scoring and ranking metrics for the three co-folding methods (AFM, AF3, and Boltz-2) was evaluated on the independent dataset to evaluate the external validation performance. The molecular docking methods were also applied to the independent dataset, and their performance was compared with that obtained on the conformation-aware PepPro dataset. This comparison was used to assess how the methods performed without the added target complexity and conformational flexibility. The performance was measured using rank-based correlation metrics (Spearman's *ρ*, Kendall's *τ*), classification-oriented metrics (ROC-AUC, PR-AUC), and early recognition (BEDROC). The detailed results are listed in Table 3. A point plot visualizing the performance of the scoring methods on the independent test set is provided in the SI (Fig. S3).

**Table 3:** Assessment of ranking correlation (Spearman *ρ*; Kendall *τ*), ranking accuracy (ROC-AUC; PR-AUC), and early recognition performance (BEDROC, *α* = 20) for two molecular docking methods and three co-folding methods on the independent dataset. All metrics were evaluated using a bootstrapped mean and a 95% confidence interval

Method metric	Mean Spearman's *ρ*	Mean Kendall's *τ*	Mean ROC-AUC	Mean PR-AUC	Mean BEDROC
**AutoDock-CrankPep**					
ADCP score	−0.64 ± 0.03	−0.43 ± 0.03	0.24 ± 0.05	0.06 ± 0.01	0.0 ± 0.0
OMM score	−0.46 ± 0.05	−0.32 ± 0.04	0.32 ± 0.05	0.09 ± 0.03	0.0 ± 0.0

**HADDOCK**					
HADDOCK score	0.29 ± 0.06	0.19 ± 0.04	**0.88** ± **0.03**	0.62 ± 0.07	0.73 ± 0.07

**AlphaFold-Multimer**					
ipTM	0.76 ± 0.02	0.55 ± 0.02	**0.89** ± **0.02**	0.70 ± 0.05	0.72 ± 0.07
ipTM + pTM	0.77 ± 0.02	**0.56** ± **0.02**	**0.90** ± **0.02**	0.71 ± 0.05	0.72 ± 0.07
Peptide pLDDT	**0.78** ± **0.02**	**0.56** ± **0.02**	**0.91** ± **0.01**	0.78 ± 0.04	0.87 ± 0.04

**AlphaFold3**					
ipTM	0.73 ± 0.02	0.52 ± 0.02	0.88 ± 0.02	**0.80** ± **0.03**	**0.93** ± **0.03**
Ranking score	0.72 ± 0.02	0.51 ± 0.02	0.86 ± 0.02	0.73 ± 0.04	0.78 ± 0.06
Peptide pLDDT	0.65 ± 0.03	0.46 ± 0.03	0.82 ± 0.02	0.73 ± 0.04	**0.90** ± **0.03**

**Boltz-2**					
ipTM	0.53 ± 0.04	0.37 ± 0.03	0.80 ± 0.02	0.72 ± 0.04	**0.90** ± **0.03**
Confidence score	0.46 ± 0.04	0.31 ± 0.03	0.75 ± 0.02	0.64 ± 0.04	0.86 ± 0.04
Peptide pLDDT	0.66 ± 0.03	0.46 ± 0.02	0.88 ± 0.02	0.77 ± 0.04	0.87 ± 0.04
Complex iPDE	0.44 ± 0.04	0.29 ± 0.03	0.77 ± 0.02	0.56 ± 0.04	0.66 ± 0.07
Complex ipLDDT	0.53 ± 0.04	0.36 ± 0.03	0.82 ± 0.02	0.71 ± 0.04	0.88 ± 0.04

ADCP performed comparatively on the independent dataset compared to the PepPro dataset. The correlations with experimental data (Spearman's *ρ* = −0.64 ± 0.03; Kendall's *τ* = 0.39 ± 0.03), low discriminative ability (ROC-AUC ≤ 0.59 ± 0.22), negligible mean precision-recall performance (PR-AUC ≈ 0.01 ± 0.01), and no early enrichment (BEDROC = 0.00).

Compared with its performance on the PepPro dataset, HADDOCK showed stronger ranking correlations on the independent dataset (Spearman's *ρ* = 0.29 ± 0.06; Kendall's *τ* = 0.19 ± 0.04), although the correlations remained weak. HADDOCK's discriminatory performance (ROC-AUC = 0.88 ± 0.03) remains among the top for all evaluated methods. HADDOCK's precision-recall and early-enrichment performance increased substantially when docked on targets where conformational change is not incorporated.

All metrics within AFM showed strong ranking correlation performance with Spearman's *ρ* ranging from 0.76 to 0.78 ± 0.02 and Kendall's *τ* ranging from 0.55 to 0.56 ± 0.02. However, peptide pLDDT showed drastic improvement based on early enrichment and precision compared to the ipTM and ipTM + pTM metrics with a PR-AUC of 0.78 ± 0.04 and a BEDROC of 0.87 ± 0.04.

AFM's ranking correlation performance on the independent dataset slightly improved compared to its performance on the PepPro dataset, especially for ipTM and ipTM + pTM (Spearman's *ρ* from 0.66–0.67 to 0.76–0.77 and a Kendall's *τ* from 0.47–0.48 to 0.55–0.56). The other metrics remained within their reported uncertainties and therefore do not indicate statistically meaningful performance differences between the two datasets, although their values were consistently lower on the PepPro dataset.

Compared with its performance on the PepPro dataset, AF3 exhibited improved relative performance compared to the other methods. ipTM achieved the highest rank correlations (Spearman's *ρ* = 0.73 ± 0.02; Kendall's *τ* = 0.52 ± 0.02) and ROC-AUC (0.88 ± 0.02), which was statistically comparable to the AF3's Ranking score (Spearman's *ρ* = 0.72 ± 0.02; Kendall's *τ* = 0.51 ± 0.02; ROC-AUC = 0.86 ± 0.02). However, its PR-AUC (0.80 ± 0.03) and BEDROC (0.93 ± 0.02) were substantially higher, highlighting stronger early recognition and precision with ipTM. Peptide pLDDT had similar early enrichment performance, but underperformed compared to ipTM in ranking correlation (Spearman's *ρ* = 0.65 ± 0.03; Kendall's *τ* = 0.46 ± 0.03) and classification performance (ROC-AUC = 0.82 ± 0.02; PR-AUC = 0.73 ± 0.04). Therefore, ipTM performed best for AF3 when performed on complexes not known to the training set.

AF3 showed a clear reduction in correlation-based ranking performance compared to AFM, indicating that the global agreement between predicted scores and the reference ranking is weaker in this setting. However, ipTM exhibited higher PR-AUC and BEDROC values, which demonstrated improved precision and early enrichment despite the reduced overall correlation.

The ranking performance of AF3 on the independent dataset improved compared to its performance on the PepPro dataset. The largest increase was observed in the correlation ranking performance, with ipTM's Spearman's *ρ* increasing from 0.54 ± 0.04 to 0.73 ± 0.02 and its Kendall's *τ* increasing from 0.38 ± 0.03 to 0.52 ± 0.02. Furthermore, the classification performance and enrichment of ipTM were also marginally better with ROC-AUC increasing from 0.76 ± 0.03 to 0.88 ± 0.02 and BEDROC increasing from 0.74 ± 0.08 to 0.93 ± 0.03.

When exposed to the independent dataset, the Boltz-2 showed distinctly different behaviour. Peptide pLDDT outperformed most metrics within Boltz-2, achieving the highest Spearman's *ρ* (0.66 ± 0.03), Kendall's *τ* (0.46 ± 0.02), ROC-AUC (0.88 ± 0.02), and PR-AUC (0.77 ± 0.04). Furthermore, it showed strong early enrichment (BEDROC = 0.87 ± 0.04), showing good prioritization of good poses in the top-ranked predictions.

Boltz-2's ipTM and complex ipLDDT early enrichment performance remained within their reported uncertainties compared to peptide pLDDT and therefore did not indicate statistically meaningful performance differences. However, ipTM and complex ipLDDT showed slightly weaker ranking correlations and reduced classification performance (ROC-AUC of 0.80–0.82 *versus* 0.88 ± 0.02 for peptide pLDDT). The confidence score further declined in rank correlation (Spearman's *ρ* = 0.46 ± 0.03). The complex iPDE exhibited the weakest performance overall, with notably low rank correlations (Spearman's *ρ* = 0.44 ± 0.04; Kendall's *τ* = 0.29 ± 0.03) and the lowest PR-AUC (0.56 ± 0.04) and BEDROC (0.66 ± 0.07).

Boltz-2 performed generally worse than AFM and AF3 on the independent set. Peptide pLDDT was competitive with the other methods based on classification and prioritization metrics. However, Boltz-2's peptide pLDDT metric showed a significantly lower ranking correlation performance than AFM's and AF3's metrics.

## Discussion

In this study, we provide a systematic evaluation of whether all-atom-based co-folding methods, like AF3 and Boltz-2, improve the accuracy and robustness of peptide co-folding compared to their residue-based predecessor AFM, and whether co-folding methods outperform state-of-the-art peptide molecular docking approaches on conformationally flexible targets.

This study revealed distinct differences between co-folding methods in their dependence on training-set representation and their ability to generalize to unseen protein–peptide complexes. Boltz-2 achieved the highest pose prediction accuracy on the PepPro dataset, which contains complexes overlapping with its training set, indicating strong performance when structurally related examples are available. Boltz-2 is trained on substantially more structures, as it includes complexes released up to 1 June 2023, whereas the training sets of AFM and AF3 are limited to structures available before 30 September 2021.^18–20^ This expanded training set likely contributes to the improved performance of Boltz-2 on the PepPro dataset.

However, this advantage did not translate to the independent dataset, where Boltz-2 showed a larger performance decrease. When the three co-folding methods were evaluated on complexes without protein- or complex-level redundancy, their performance decreased substantially to comparable levels, with Boltz-2 performing slightly below AFM and AF3. These results indicate that the substantial performance increase of Boltz-2 on the PepPro dataset is strongly associated with the representation of related complexes in its training data.

In contrast, AFM and AF3 exhibited smaller reductions in predictive performance on the independent dataset. AFM experienced the smallest reduction and performed best on complex-level non-redundant complexes, providing a slightly stronger correlation performance. AF3 showed the best enrichment and accuracy performance. Together, these findings indicate that AFM and AF3 have a slightly more robust ability to generalize to previously unseen protein–peptide complexes, whereas Boltz-2 demonstrates particularly strong performance in reproducing structural interaction patterns represented in its training set. These differences may be particularly relevant in the context of *de novo* binder design. Because designed peptides are unique and are unlikely to be represented in the internal training data, AFM and AF3 may more reliably predict true binding poses in scenarios where prior structural information is limited.

Training-set representation is known to influence pose prediction outcomes through bias, which can lead to misleading performance assessments in *de novo* binder design.^50^ However, depending on the context, this can also be advantageous. This study provides a clear case example. The PepPro dataset includes the PDB entries 3BEF and 3LU9, both of which contain an extracellular fragment of proteinase-activated receptor 1 (PAR1) in complex with prothrombin. According to EMBL-EBI PDBe-KB, more than 250 PDB structures of prothrombin have been reported, encompassing a diverse range of binding motifs.^51^ In 3BEF and 3LU9, prothrombin is co-crystallized with PAR1 at distinct binding motifs, requiring co-folding methods to prioritize the correct motif for each peptide to obtain an accurate prediction relative to the reference pose. AFM and AF3 positioned both peptides within the same binding motif, whereas Boltz-2 correctly identified the binding sites in both instances. Because both PDB entries were included in the training sets of the evaluated co-folding methods, this example demonstrates how training-set representation can influence pose prediction outcomes differently depending on the method used. Notably, despite identical training-set exposure, only Boltz-2 correctly resolved both binding modes. This indicates that, although it is strongly influenced by training data, it can still discriminate between distinct binding motifs, whereas AFM and AF3 appear biased toward a single binding site.

Molecular docking methods showed a clearer reduction in pose-prediction success as the conformational difference between the apo and holo protein structures increased. When the apo structure differs substantially from the peptide-bound conformation, the binding pocket may not have the geometry required to accommodate the peptide, thereby limiting the ability of the docking algorithm to identify a near-native pose. A previous benchmark by Weng *et al.*, in which ADCP was evaluated on the PepSet dataset, reported similar performance on a “difficult” protein subset, consistent with our findings and highlighting ADCP's limitations in handling flexibility.^28^ In that study, “difficult” was defined as cases where the bound peptide adopts a conformation with high RMSD relative to its unbound (ideal) state.

In contrast, the co-folding methods were less sensitive to increasing apo–holo conformational differences. Because these methods predict the protein–peptide complex directly from sequence, they are not constrained by a fixed experimental receptor conformation and may implicitly model structural rearrangements associated with peptide binding. This may provide an advantage for targets that undergo substantial conformational changes upon complex formation. However, it is important to consider that the observed performance may have been inflated by data leakage. This highlights the need for a benchmark dataset containing conformationally distinct apo–holo complexes that are non-redundant with respect to the training data.

HADDOCK showed that molecular docking can still be a valuable tool for identifying high-quality binding poses on conformationally flexible targets, even within ranking sets that remain feasible for chemical synthesis, such as 20 candidates. Our research shows that, within the top 20 predictions, the pose prediction performance almost reaches the level of the co-folding methods, even when there are significant conformational changes between the apo and holo states. Moreover, HADDOCK regularly obtained acceptable- and medium-quality poses, indicating that useful binding conformations could still be identified despite its lower overall top-ranked pose accuracy. Similarly, Weng *et al.* showed that HADDOCK had good early enrichment and ranking performance, indicated by its higher success rate in the “difficult” subset, suggesting that HADDOCK is equipped to successfully dock not only flexible proteins but also flexible peptides.^28^

When docked on a known conformational holo-state, HADDOCK outperformed the co-folding methods substantially. This highlights the value of HADDOCK when an experimentally determined holo structure, or a reliable holo-like model, is available or elucidated. Furthermore, HADDOCK is still actively being supported and improved, with the latest update in 2024.^52^ Therefore, initial prediction of a holo-state pose using confidence-based co-folding, followed by HADDOCK's physics-based docking, could provide a valuable complementary strategy for improving pose prediction accuracy.

These differences in generalization and prediction accuracy have important implications for peptide binder design workflows. State-of-the-art peptide and mini-protein binder generators, like RFpeptides and BindCraft, still filter the bulk of their hits using AlphaFold-based architectures.^53,54^ There are several possible explanations for this behaviour, including legal constraints associated with AF3, which may complicate ownership and intellectual property claims. However, the primary explanation is that the AFM architecture remains highly robust, as demonstrated on the independent dataset. Boltz-2 may nevertheless be valuable for certain proteins, especially when experimentally determined structures are available. For example, BoltzGen is currently in development, which will apply Boltz-2 for protein and peptide design.^55^ A generative binder design pipeline that integrates multiple models, not limited to only Boltz-2 or AFM, and capitalizes on their respective strengths may offer substantial benefits for advancing peptide binder design across a broader range of protein targets.

Knowing which internal metrics should be used in methods plays a critical role in identifying accurate peptide binding poses and prioritizing candidates for experimental validation. Therefore, we evaluated the behaviour of commonly used confidence and ranking metrics independently of the prediction methods to contextualize structural pose prediction performance. The ipTM is widely applied to assess protein–protein and protein–peptide complex accuracy.^56,57^ A study by Lee *et al.* has suggested that ipTM performs particularly well for short polypeptide fragments compared to full-length protein complexes.^58^ In agreement with these observations, in our study, ipTM demonstrated robust ranking behaviour on the independent dataset. Notably, AF3's ipTM showed strong early enrichment performance, indicating its suitability for prioritizing a limited number of candidate binders in practical design workflows.

In contrast to ipTM, peptide-specific pLDDT scores have not been systematically evaluated for protein–peptide complexes. Prior benchmarking studies have shown that interface residue-based pLDDT can assist in model selection for protein–protein interactions.^59,60^ Extending this concept to peptide complexes, peptide pLDDT demonstrated consistent and robust performance across methods. In particular, AFM's peptide pLDDT exhibited generally the strongest correlation with the DockQ scores, suggesting that it reliably reflects pose correctness rather than global complex confidence.

Taken together, these findings demonstrate that combining ipTM and peptide pLDDT may further improve pose selection accuracy due to their complementary but distinct performance on protein–peptide prediction quality. ipTM appears particularly effective for ranking and early enrichment, whereas peptide pLDDT provides a reliable indicator of structural agreement with experimentally determined binding poses. Integrating both metrics into a combined ranking framework may therefore improve discrimination between near-native and non-native poses. Future work should systematically explore multi-metric scoring strategies to enhance robustness in peptide binder design.

Several limitations should be considered when interpreting these results. Peptide docking and co-folding were performed on complexes that can exist in different and more complex conformations. The peptides used in the datasets could, depending on crystallization conditions, represent fragments of larger structures or proteins rather than biologically characterized peptides. During the construction of the independent test set, the stringent global TM-score cutoff may have excluded complexes with distinct peptide-binding interfaces, thereby reducing the size and structural diversity of the test set. In addition, experimentally determined apo structures were not retrieved for the independent test set complexes. Consequently, the effects of training-data leakage and protein conformational changes could not be fully disentangled. Future studies should therefore include experimentally determined apo structures for the complexes in the independent test set. This would enable the effects of training-data leakage to be distinguished from those of apo-to-holo conformational changes and provide a more direct assessment of method generalization on flexible targets.

Future work should focus on integrating complementary co-folding models and confidence metrics into unified prediction pipelines, enabling robust peptide binder design across both well-characterized targets and novel protein–peptide interaction landscapes.

## Conclusion

This study demonstrates that co-folding performance decreases substantially on complexes structures that are non-redundant with the training set, particularly when peptide information is not available. Nevertheless, co-folding methods outperform peptide-docking approaches on distinct conformationally apo–holo changes. Peptide-only pLDDT correlates strongly with structural accuracy and complements ipTM, which supports robust ranking and early enrichment. These findings highlight that the selection of computational methods and ranking metrics should be aligned with the intended study outcomes to support robust peptide design.

## Author contributions

M. F.: conceptualization, data curation, methodology, software, data analysis, writing and editing. W. J.: conceptualization, methodology, software, review and editing, supervision. N. B.: conceptualization, methodology, software, review and editing. P. K.: conceptualization, methodology, review and editing, supervision. A. V.: conceptualization, review and editing, supervision. G. W.: conceptualization, methodology, software, review and editing, supervision.

## Conflicts of interest

The authors declare no competing and financial interests.

## Data Availability

The data supporting this article have been included as part of the supplementary information (SI). The SI and the non-redundant independent test set are available at https://zenodo.org/records/21706081. Supplementary information: an overview of the benchmarked complexes, the phylogenetic tree, a comprehensive overview of all success rates, and point plots of all scoring metrics. See DOI: https://doi.org/10.1039/d6dd00242k.
